# Attention-deficit hyperactivity disorder diagnoses and prescriptions in UK primary care, 2000–2018: population-based cohort study

**DOI:** 10.1192/bjo.2023.512

**Published:** 2023-07-17

**Authors:** Douglas G. J. McKechnie, Elizabeth O'Nions, Sandra Dunsmuir, Irene Petersen

**Affiliations:** Department of Primary Care and Population Health, University College London, UK; Division of Psychology and Language Sciences, Faculty of Brain Sciences, University College London, UK

**Keywords:** Attention-deficit hyperactivity disorders, primary care, central nervous system stimulants, epidemiology, socioeconomic deprivation

## Abstract

**Background:**

Rates of diagnosed attention-deficit hyperactivity disorder (ADHD) may be increasing in the UK.

**Aims:**

Estimate incidence and prevalence of ADHD diagnoses and ADHD prescriptions in UK adults and children in primary care.

**Method:**

We conducted a cohort study using IQVIA Medical Research Data, a UK primary care database. Rates of ADHD diagnoses and ADHD prescriptions were calculated between 2000 and 2018 for individuals aged 3–99 years, analysed by age, gender, social deprivation status and calendar year.

**Results:**

Of 7 655 931 individuals, 35 877 (0.5%) had ADHD diagnoses; 18 518 (0.2%) received ADHD medication prescriptions. Diagnoses and prescription rates were greater in men versus women, children versus adults, and deprivation status (nearly double in most deprived versus least deprived quintile). By 2018, the proportion of ADHD diagnoses was 255 per 10 000 (95% CI 247–263) in boys and 67.7 per 10 000 (95% CI 63.5–71.9) in girls; for adults, it was 74.3 per 10 000 (95% CI 72.3–76.2) in men and 20 per 10 000 (95%CI 19.0–21.0) in women. Corresponding figures for prescriptions were 156 per 10 000 (95% CI 150–163) in boys, 36.8 per 10 000 (95% CI 33.8–40.0) in girls, 13.3 per 10 000 (95% CI 12.5–14.1) in men and 4.5 per 10 000 (95% CI 4.1–5.0) in women. Except among 3- to 5-year-olds, the incidence and prevalence of ADHD diagnoses and prescriptions have increased from 2000 to 2018 in all age groups. The absolute increase was highest in children, but the relative increase was largest among adults (e.g. among men aged 18–29 years, approximately 20-fold and nearly 50-fold increases in diagnoses and prescriptions, respectively).

**Conclusions:**

The incidence and prevalence of both ADHD diagnoses and medication are highest among children. Proportionally, rates increased most among adults during 2000–2018. ADHD diagnoses and prescriptions are associated with socioeconomic deprivation.

Attention-deficit hyperactivity disorder (ADHD) is a neurodevelopmental disorder, characterised by symptoms of inattention, hyperactivity and impulsivity, with onset in childhood.^1^ In high-income countries, the prevalence of diagnosed ADHD in both children and adults has steadily increased over the past few decades,^2^ but prevalence rates of diagnosis and ADHD medication use vary hugely between countries; for example, the prevalence of ADHD medication use in the entire adult population in 2010 ranged from <0.01% in Japan to 1.42% in the USA.^2^ Most studies of the incidence and prevalence of ADHD and ADHD medication use in the UK have focused on children and adolescents,^3–5^ with three studies also including adults.^2,6,7^ An analysis of data from 14 countries collected between 2001 and 2015 reported that rates of ADHD medication prescribing have increased.^2^ ADHD is strongly associated with socioeconomic deprivation in children,^4,8,9^ although none of the three UK-based population studies examining adult ADHD have examined this association in adulthood.^2,6,7^

Understanding the epidemiology of ADHD is important for service planning, education and resource allocation. Measuring ADHD diagnoses, as well as ADHD prescriptions, is important because ADHD is sometimes managed non-pharmacologically. The aim of this study was to provide estimates of rates of ADHD diagnoses and prescriptions of ADHD medication in UK primary care, both new and ongoing, between 2000 and 2018. We also aimed to describe how these rates differ by age, gender and socioeconomic deprivation status, in children, adolescents and adults.

## Method

### Study design

This was a population-based cohort study, using data from IQVIA Medical Research Data (IMRD). IMRD incorporates data from The Health Improvement Network (THIN), a Cegedim database. Reference made to THIN is intended to be descriptive of the data asset licensed by IQVIA.

Our study examines incidence and prevalence based on diagnoses and prescriptions coded in primary care records, which is likely to be lower than the prevalence of people with ADHD symptoms in the community. We therefore favour the terms ‘new ADHD diagnoses/prescriptions’ in place of incidence, and ‘proportion of people with ADHD diagnoses/prescribed ADHD medication’ rather than prevalence in this paper, although the operational definition is the same. ‘New ADHD diagnoses’ refer to the time the diagnosis is made and not the onset of symptoms, which cannot be estimated from primary care records.

IMRD holds ethical approval to collect and supply data for research purposes from the NHS London – South East Research Ethics Committee (reference number 18/LO/0441). This study was granted approval by IMRD's Scientific Review Committee in January 2022 (reference number 21SRC062).

### Setting

IMRD contains de-identified data drawn from routinely collected primary healthcare records. Data from approximately 12 million people are included in the database. Information collection begins from when each person registers with an IMRD-affiliated general practice. The IMRD sample is generally representative of the whole UK primary care population.^10^ Almost all of the UK population are registered with a primary care practice.^11^ The database includes diagnoses coded with the Read Code system,^12^ and all prescriptions issued by primary care clinicians. IMRD also contains information on year of birth, gender and social deprivation status. Based on individuals’ residential postcode linked to population census data from 2001, deprivation is estimated by using the Townsend score, a combined measure of unemployment, car ownership, home ownership and household overcrowding.^13^ The scores are defined for areas of around 150 households, and grouped into quintiles.

In the UK, ADHD is diagnosed by psychiatrists or paediatricians in secondary care. Initiation and stabilisation of medication for ADHD should additionally be performed by secondary care.^14^ However, once this is completed, the responsibility for ongoing prescription and physical health monitoring is usually transferred to primary care practices, under a ‘shared-care’ agreement.^14^ This system, however, is not seamlessly implemented across the UK. There are service gaps in ADHD care, particularly for adults, and shared-care agreements can be precarious.^15^ Diagnosis and treatment may also be made in the private sector. There is an incentive for patients to seek shared care for ADHD medications between their private psychiatrist and National Health Service (NHS) primary care provider (ADHD medications are substantially cheaper as NHS, rather than private, prescriptions), but many primary care clinicians are reluctant to engage into shared-care agreements with a private provider.^15^

### Study population

This study used anonymised data from electronic health records; individual consent was therefore not applicable. All data used in this study was taken from primary care practices that fulfilled both criteria of acceptable mortality recording^16^ and acceptable computer usage.^17^ Both are measures of quality assurance. General practices and individuals missing data on Townsend deprivation quintile were excluded, removing 138 practices. A further seven practices supplied less than a full calendar year of eligible data and were removed, leaving 649 practices from which the study population was drawn.

For calculations of new diagnoses/prescriptions, we included all individuals aged 3–99 years who were permanently registered at a participating general practice between 1 January 2000 and 31 December 2018. ADHD diagnoses are rarely made in children under 3 years of age.^18^ The primary outcome of interest was a ‘new’ diagnosis of ADHD, or a first prescription of ADHD medication in primary care. When calculating rates of new diagnoses/prescriptions, we excluded individuals with an ADHD diagnosis or an ADHD prescription before start of follow-up (see ‘Statistical analysis’ section for details of follow-up), or within 6 months of registering at a general practice, as these were more likely to reflect coding of pre-existing diagnoses or the continuation of prescriptions initially prescribed at another practice.^19^

To calculate the proportion of people with ADHD diagnoses or with prescriptions for ADHD medication, all individuals with at least one full calendar year of data in the study period were included.

### Definition of main outcomes

A list of Read Codes indicating an ADHD diagnosis was constructed with established methods;^20^ the full list is given in Supplementary Table 1, available at https://doi.org/10.1192/bjo.2023.512. Any individual with their first record of an ADHD diagnosis occurring during the follow-up period was defined as a new case; the time of first diagnosis was taken as the earliest recorded date of a qualifying code.

A new prescription was defined as the first recorded prescription during the follow-up period of any of the following: methylphenidate, dexamfetamine, dexamfetamine with amfetamine, lisdexamfetamine, atomoxetine and guanfacine. Methylphenidate and dexamfetamine also have a UK product licence for use in narcolepsy. Individuals with a prescription for ADHD medication, a recorded diagnosis of narcolepsy at any time (Read Code F271.00) and no recorded ADHD diagnosis at any time, were treated as medication non-users when reporting prescription rates/proportions.

For proportion calculations, individuals’ date of diagnosis was taken as the earliest recorded date of a qualifying code; if this occurred before cohort entry, they were treated as having ADHD diagnoses from the entry date onward. Ongoing prescription of ADHD medication was defined as at least two prescriptions within a year of one another, for any of the medicines listed above.

### Statistical analysis

Rates of ADHD diagnoses and medication were analysed separately for children (aged 3–17 years) and adults (aged 18–99 years).

Rates of new ADHD diagnoses and first-time ADHD prescriptions were estimated per 100 000 person-years at risk (PYAR) as the total number of new ADHD diagnoses or first-time ADHD medication prescriptions recorded between 2000 and 2018, divided by the total number of person-years of follow-up. Rates of new ADHD diagnosis and ADHD prescriptions were additionally estimated by age, gender, Townsend score and calendar year.

The proportion of ADHD diagnoses and prescriptions was determined by dividing the number of individuals with an existing record of a diagnosis or a prescription by the total number of individuals in the eligible cohort.

Exploratory analyses demonstrated a significant interaction between age and gender; therefore, all analyses were stratified by gender.

Poisson regression was performed to obtain confidence intervals for crude incidence and prevalence rates. Multivariable Poisson regression models, with (log)person-time as an offset, were used to determine incidence rate ratios, adjusting for age group and Townsend deprivation score. Likewise, prevalence risk ratios were estimated by adjusting for age and Townsend deprivation scores. Multilevel random intercept models were used to adjust for the effect of clustering by general practice.

Statistical analysis was performed with Stata version 17 for Windows (StataCorp, College Station, Texas, USA).

### ADHD medication prescriptions without ADHD diagnoses

Prior work has reported that ADHD medication is frequently prescribed to people without a recorded ADHD diagnosis.^21^ We analysed the subgroup of those who received ADHD medication without an ADHD diagnosis in our data-set, by examining all clinical codes recorded within 2 years of their first ADHD medication prescription, to evaluate other possible indications for the prescriptions.

## Results

**Fig. 1:**
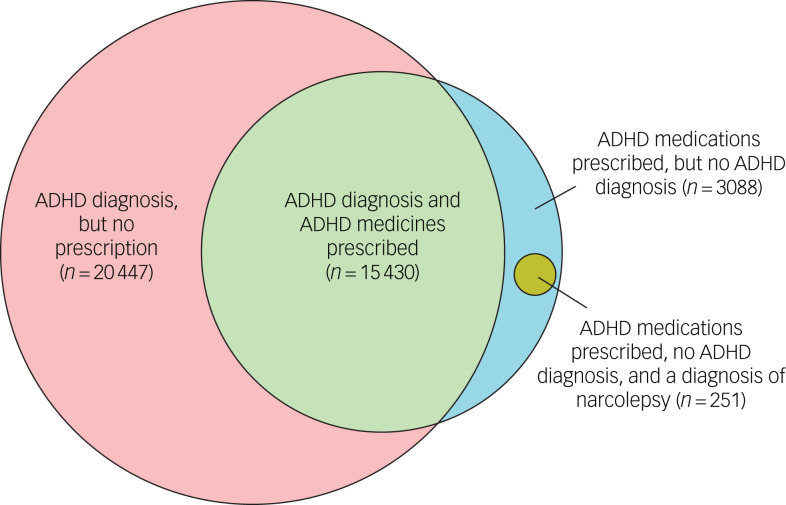
Venn diagram of the relationship between individuals with ADHD diagnoses, individuals with ADHD medication usage and individuals with narcolepsy; ADHD medication usage and no ADHD diagnosis. ADHD, attention-deficit hyperactivity disorder.

In total, 7 655 931 individuals contributed at least one full calendar year of data. Of these, 35 877 individuals (0.5%) had a diagnosis of ADHD, and 18 518 (0.2%) individuals received at least two prescriptions within a year for ADHD medications from primary care. A total of 3088 people were prescribed ADHD medications, but did not have a recorded diagnosis of ADHD; of these, 251 (8%) had a diagnosis of narcolepsy (Fig. 1). Those with a narcolepsy diagnosis and no concurrent ADHD diagnosis were treated as ADHD medication non-users in the following analyses. Of the 35 877 individuals with an ADHD diagnosis, 20 447 (57%) did not receive ADHD medication prescriptions from primary care.

### New ADHD diagnoses and prescriptions

In children, new diagnosis rates were highest between 6 and 9 years of age, and in adults, new diagnosis rates were highest between 18 and 29 years of age (Table 1). New diagnosis rates were highest in the most deprived areas for both children and adults (approximately two- to three-fold higher in the most deprived quintile versus the least deprived quintile) (Table 1). Similar patterns were seen for first prescriptions of ADHD medication use (Table 2).

**Table 1:** Rates of new attention-deficit hyperactivity disorder diagnoses by age group and Townsend deprivation quintile in males and females, presented separately for adults and children

	Male		Female	
	Rate per 100 000 PYAR (95% CI)	Adjusted^a^ incidence rate ratio (95% CI)	Rate per 100 000 PYAR (95% CI)	Adjusted^a^ incidence rate ratio (95% CI)
Children (3–17 years)				
Age group, years				
3–5	106.53 (100.68–112.62)	0.26 (0.24–0.27)	22.17 (19.48–25.12)	0.23 (0.20–0.26)
6–9	409.56 (399.54–419.77)	1	95.9 (90.94–101.06)	1
10–15	212.39 (206.44–218.46)	0.52 (0.51–0.54)	52.26 (49.21–55.46)	0.55 (0.51–0.59)
16–17	57.11 (51.81–62.81)	0.14 (0.13–0.15)	23.13 (19.6–27.12)	0.24 (0.20–0.28)
Townsend deprivation quintile				
1	149.34 (143.1–155.79)	1	37.16 (33.94–40.61)	1
2	172.91 (165.51–180.55)	1.19 (1.12–1.27)	44.01 (40.16–48.13)	1.23 (1.08–1.40)
3	230.79 (222.32–239.51)	1.59 (1.50–1.69)	53.24 (49.05–57.68)	1.49 (1.32–1.69)
4	296.34 (286.22–306.73)	2.08 (1.97–2.21)	70.96 (65.88–76.33)	2.05 (1.82–2.32)
5	325.13 (312.73–337.9)	2.49 (2.33–2.65)	78.2 (72.06–84.72)	2.46 (2.15–2.80)
Adults (18–99 years)				
Age group, years				
18–29	16.45 (15.31–17.66)	1	8.82 (7.97–9.74)	1
30–39	8.87 (8.04–9.77)	0.54 (0.48–0.61)	6.05 (5.36–6.81)	0.71 (0.60–0.82)
40–49	4.6 (4.03–5.23)	0.29 (0.25–0.33)	4.21 (3.66–4.82)	0.51 (0.43–0.60)
≥50	2.4 (2.13–2.7)	0.15 (0.13–0.18)	2.56 (2.3–2.85)	0.31 (0.27–0.36)
Townsend deprivation quintile				
1	4.31 (3.83–4.84)	1	3.09 (2.69–3.53)	1
2	5.19 (4.62–5.81)	1.19 (1.01–1.40)	3.79 (3.31–4.31)	1.19 (0.99–1.44)
3	6.45 (5.81–7.15)	1.33 (1.13–1.56)	4.23 (3.72–4.79)	1.24 (1.03–1.50)
4	8.9 (8.08–9.79)	1.70 (1.45–1.99)	5.59 (4.96–6.29)	1.53 (1.27–1.85)
5	9.67 (8.64–10.79)	1.82 (1.53–2.16)	7.39 (6.5–8.37)	2.00 (1.64–2.44)

PYAR, person-years at risk.

a. Adjusted for age group and Townsend deprivation quintile.

**Table 2:** Rates of first prescriptions for attention-deficit hyperactivity disorder medications, by age group and Townsend deprivation quintile in males and females, presented separately for adults and children

	Male		Female	
	Rate per 100 000 PYAR (95% CI)	Adjusted incidence rate ratio (95% CI)^a^	Rate per 100 000 PYAR (95% CI)	Adjusted incidence rate ratio (95% CI)^a^
Children (3–17 years)				
Age group, years				
3–5	37.61 (34.17–41.3)	0.11 (0.10–0.12)	7.15 (5.66–8.91)	0.10 (0.08–0.12)
6–9	337.71 (328.64–346.97)	1	73.24 (68.92–77.77)	1
10–15	214.57 (208.61–220.65)	0.64 (0.62–0.67)	49.69 (46.72–52.81)	0.68 (0.62–0.74)
16–17	55.1 (49.91–60.68)	0.16 (0.15–0.18)	20.06 (16.78–23.78)	0.27 (0.23–0.32)
Townsend deprivation quintile				
1	125.71 (120–131.62)	1	31.5 (28.54–34.69)	1
2	147.28 (140.48–154.33)	1.20 (1.12–1.28)	35.61 (32.16–39.34)	1.16 (1.01–1.34)
3	200.33 (192.46–208.44)	1.67 (1.56–1.78)	43.04 (39.29–47.05)	1.41 (1.23–1.62)
4	254.1 (244.76–263.7)	2.18 (2.05–2.33)	56.75 (52.22–61.56)	1.95 (1.70–2.23)
5	274.53 (263.19–286.24)	2.55 (2.38–2.73)	59.28 (53.96–64.98)	2.16 (1.86–2.51)
Adults (18–99 years)				
Age group, years				
18–29	16.32 (15.19–17.52)	1	7.69 (6.9–8.55)	1
30–39	7.56 (6.8–8.39)	0.46 (0.41–0.52)	4.66 (4.06–5.33)	0.62 (0.52–0.74)
40–49	3.76 (3.25–4.33)	0.23 (0.20–0.27)	2.74 (2.3–3.23)	0.37 (0.31–0.46)
≥50	1.64 (1.41–1.88)	0.10 (0.09–0.12)	1.27 (1.08–1.48)	0.17 (0.14–0.21)
Townsend deprivation quintile				
1	3.74 (3.3–4.24)	1	2.13 (1.8–2.51)	1
2	4.64 (4.1–5.22)	1.18 (0.99–1.40)	2.64 (2.24–3.08)	1.19 (0.95–1.49)
3	6.36 (5.72–7.05)	1.47 (1.25–1.74)	3.29 (2.84–3.79)	1.38 (1.10–1.72)
4	7.02 (6.29–7.81)	1.48 (1.25–1.76)	3.93 (3.4–4.52)	1.51 (1.21–1.89)
5	8.72 (7.75–9.78)	1.76 (1.47–2.12)	4.91 (4.19–5.71)	1.82 (1.43–2.31)

PYAR, person-years at risk.

a. Adjusted for age group and Townsend deprivation quintile.

**Fig. 2:**
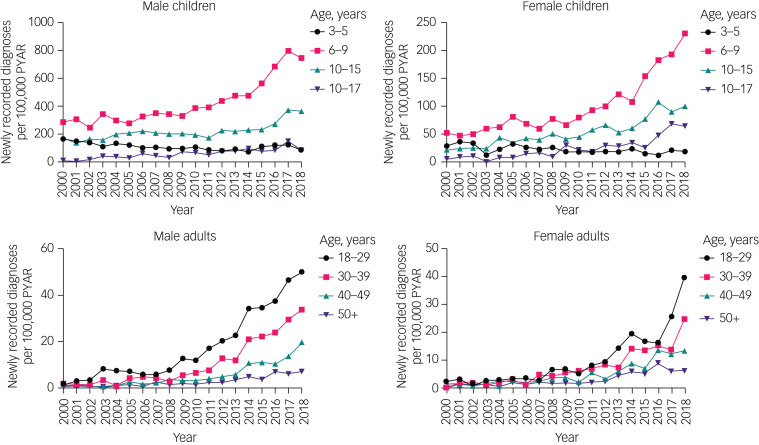
Time trends of new diagnoses in children and adults, by gender and age group. Note different *y*-axis scales for each group. Raw data are given in Supplementary Table 2. PYAR, person-years at risk.

The overall rate of new ADHD diagnoses increased from 2000 to 2018 in both males (the rate doubled in those aged under 18 years and increased nearly 20-fold in those aged over 18 years) and females (the rate quadrupled in those aged under 18 years and increased 15-fold in those aged over 18 years) (Fig. 2, Supplementary Table 2).

Although all other age groups showed an increase in rates of new diagnoses, in the 3–5 year age group, rates declined between 2000 and 2018, decreasing by about 50% in boys and about 33% in girls.

There was evidence of a changing gender ratio in the rates of new diagnoses across the age groups. In those aged 3–15 years, rates of new diagnoses were approximately four times higher in males than in females. This decreased to an approximate 2:1 ratio for 16–29 years, a 1.5:1 ratio for 30–39 years and an approximate 1:1 ratio for ≥40 years.

**Fig. 3:**
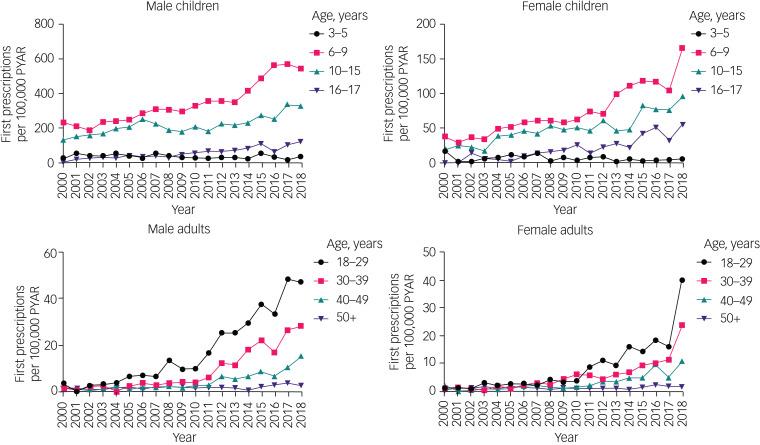
Time trends of first prescriptions in children and adults, by gender and age group. Note different *y*-axis scales for each group. Raw data are given in Supplementary Table 2. PYAR, person-years at risk.

First prescription rates also increased from 2000–2018 in both gender (the rate doubled in male children, increased eight-fold in male adults, increased over four-fold in female children, and increased 20-fold in female adults) (Fig. 3, Supplementary Table 2).

First prescription rates increased in all age groups except in children aged 3–5 years, where rates remained relatively low throughout the study period (boys: from 26.4 per 100 000 PYAR (95% CI 12.7–48.6) to 36.7 per 100 000 PYAR (95% CI 18.9–64.1); girls: from 17.6 per 100 000 PYAR (95% CI 6.5–38.3) to 6.5 per 100 000 PYAR (95% CI 0.8–23.3)).

### Prevalent ADHD diagnoses and prescriptions

The overall proportion of ADHD diagnoses in male children aged 3–17 years was 175 per 10 000 (95% CI 174–177), or 1.8%; in female children aged 3–17 years, it was 37.7 per 10 000 (95% CI 37.1–38.3), or 0.4%. For male adults aged 18–99 years, the overall proportion was 28.8 per 10 000 (95% CI 28.6–29.0), or 0.3%; for female adults aged 18–99 years, it was 7.2 per 10 000 (95% CI 7.1–7.3), or 0.07%.

The proportion of ADHD medication use for male children was 90.3 per 10 000 (95% CI 89.5–91.2), or 0.9%; and for female children it was 17.1 per 10 000 (95% CI 16.8–17.5), or 0.2%. In male adults, the proportion of ADHD medication use was 4.1 per 10 000 (95% CI 4.0–4.2), or 0.04%; and in female adults, it was 1.4 per 10 000 (95% CI 1.4–1.5), or 0.01%.

The proportions of both diagnoses and medication use were highest among young people aged 10–15 years and 16–17 years, and higher in areas of greater deprivation (approximately two-fold higher in the most deprived quintile than in the least deprived quintile) (Tables 3 and 4). The prevalence of ADHD medication usage peaked in the 10–15 year age group, and decreased in older age groups.

**Table 3:** Proportions of people with attention-deficit hyperactivity disorder diagnoses, by age group and Townsend deprivation quintile in males and females, presented separately for children and adults

	Male		Female	
	Diagnostic prevalence per 10 000 (95% CI)	Adjusted prevalence ratio (95% CI)^a^	Diagnostic prevalence per 10 000 (95% CI)	Adjusted prevalence ratio (95% CI)^a^
Children (3–17 years)				
Age group, years				
3–5	18.1 (17.13–19.1)	0.15 (0.14–0.16)	4.44 (3.96–4.97)	0.15 (0.14–0.17)
6–9	121.31 (119.51–123.12)	1	28.51 (27.62–29.43)	1
10–15	240.5 (238.43–242.58)	2.00 (1.96–2.03)	51.07 (50.07–52.09)	1.80 (1.73–1.87)
16–17	250.44 (246.76–254.15)	2.06 (2.03–2.11)	52.78 (50.98–54.63)	1.83 (1.75–1.92)
Townsend deprivation quintile				
1	121.27 (119.36–123.2)	1	5.29 (5.11–5.47)	1
2	139.55 (137.29–141.83)	1.18 (1.15–1.20)	5.29 (5.1–5.48)	1.20 (1.12–1.26)
3	178.34 (175.8–180.9)	1.51 (1.48–1.55)	6.92 (6.7–7.14)	1.51 (1.43–1.59)
4	231.83 (228.78–234.9)	1.98 (1.94–2.03)	9.35 (9.07–9.64)	2.06 (1.95–2.16)
5	247.48 (243.81–251.2)	2.31 (2.26–2.37)	12.08 (11.69–12.47)	2.31 (2.12–2.44)
Adults (18–99 years)				
Age group, years				
18–29	134.41 (133.31–135.52)	1	26.55 (26.04–27.07)	1
30–39	17.37 (16.97–17.77)	0.13 (0.13–0.13)	6.19 (5.96–6.44)	0.24 (0.23–0.25)
40–49	6.47 (6.24–6.7)	0.05 (0.05–0.05)	4.24 (4.06–4.43)	0.16 (0.16–0.17)
≥50	2.31 (2.22–2.4)	0.02 (0.02–0.02)	2.54 (2.45–2.63)	0.10 (0.10–0.10)
Townsend deprivation quintile				
1	18.81 (18.47–19.15)	1	25.71 (24.79–26.65)	1
2	21.15 (20.76–21.54)	1.08 (1.06–1.11)	29.61 (28.52–30.72)	0.97 (0.92–1.02)
3	29.56 (29.09–30.03)	1.35 (1.32–1.38)	37.24 (36.04–38.48)	1.15 (1.10–1.21)
4	39.1 (38.52–39.69)	1.63 (1.59–1.68)	50.69 (49.2–52.2)	1.42 (1.36–1.50)
5	46.76 (46–47.54)	1.96 (1.90–2.01)	53.62 (51.86–55.42)	1.69 (1.60–1.78)

a. Adjusted for age group and Townsend deprivation score.

**Table 4:** Proportions of people with attention-deficit hyperactivity disorder medication prescriptions, by age group and Townsend deprivation quintile in males and females, presented separately for children and adults

	Male		Female	
	Medication prevalence per 10 000 (95% CI)	Adjusted prevalence ratio (95% CI)^a^	Medication prevalence per 10 000 (95% CI)	Adjusted prevalence ratio (95% CI)^a^
Children (3–17 years)				
Age group, years				
3–5	2.21 (1.88–2.58)	0.03 (0.03–0.04)	0.39 (0.26–0.57)	0.03 (0.02–0.04)
6–9	63.9 (62.6–65.21)	1	13.06 (12.45–13.68)	1
10–15	135.77 (134.22–137.33)	2.15 (2.10–2.20)	25.29 (24.59–26.01)	1.95 (1.85–2.06)
16–17	96.33 (94.05–98.64)	1.51 (1.47–1.56)	19.04 (17.97–20.17)	1.45 (1.35–1.56)
Townsend deprivation quintile				
1	59.3 (57.97–60.65)	1	12.03 (11.41–12.68)	1
2	66.25 (64.7–67.83)	1.13 (1.09–1.17)	13.42 (12.69–14.18)	1.17 (1.08–1.26)
3	92.33 (90.51–94.17)	1.61 (1.56–1.66)	16.88 (16.07–17.71)	1.47 (1.36–1.59)
4	121.72 (119.52–123.95)	2.18 (2.11–2.24)	22.67 (21.69–23.69)	2.00 (1.86–2.16)
5	136.89 (134.16–139.66)	2.70 (2.61–2.80)	24.42 (23.24–25.64)	2.33 (2.15–2.53)
Adults (18–99 years)				
Age group, years				
18–29	18.21 (17.8–18.62)	1	4.73 (4.51–4.95)	1
30–39	2.43 (2.29–2.59)	0.13 (0.13–0.14)	1.45 (1.33–1.57)	0.31 (0.28–0.34)
40–49	1.42 (1.32–1.53)	0.08 (0.07–0.09)	1.12 (1.02–1.22)	0.24 (0.22–0.27)
≥50	0.45 (0.41–0.49)	0.03 (0.02–0.03)	0.45 (0.41–0.48)	0.10 (0.09–0.11)
Townsend deprivation quintile				
1	2.68 (2.56–2.81)	1	0.99 (0.91–1.07)	1
2	2.59 (2.46–2.73)	0.91 (0.85–0.98)	1.14 (1.05–1.23)	1.08 (0.96–1.20)
3	4.41 (4.23–4.6)	1.42 (1.33–1.51)	1.4 (1.3–1.5)	1.20 (1.07–1.34)
4	5.62 (5.4–5.84)	1.63 (1.52–1.74)	1.74 (1.62–1.87)	1.39 (1.24–1.56)
5	6.84 (6.55–7.14)	1.98 (1.84–2.13)	2.28 (2.11–2.45)	1.77 (1.57–2.01)

Prescribed medications included were dexamfetamine, lisdexamfetamine, methylphenidate, guanfacine and atomoxetine.

a. Adjusted for age group and Townsend deprivation score.

**Fig. 4:**
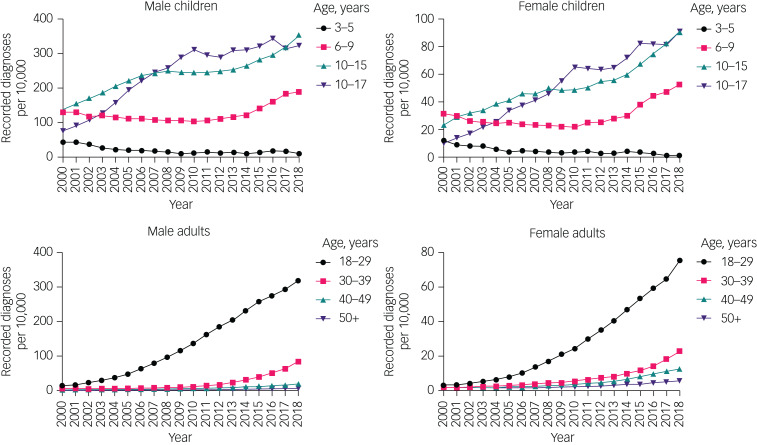
Time trends of the proportions of attention-deficit hyperactivity disorder diagnoses in children and adults, by gender and age group. Note different *y*-axis scales for each group. Raw data are given in Supplementary Table 3.

Overall, the proportion of ADHD diagnoses during 2000–2018 increased in children (it approximately doubled for boys and trebled for girls) and, proportionally, more so in adults (it increased over 18-fold in men and over ten-fold in women) (Fig. 4, Supplementary Table 3).

The proportion of ADHD diagnoses increased over time in all age groups, except the 3–5 year age group, in which the proportion declined. The proportion of children with ADHD diagnoses aged 6–9 years did not substantially change between 2000 and 2010, but increased thereafter, with an overall increase between 2000 and 2018.

**Fig. 5:**
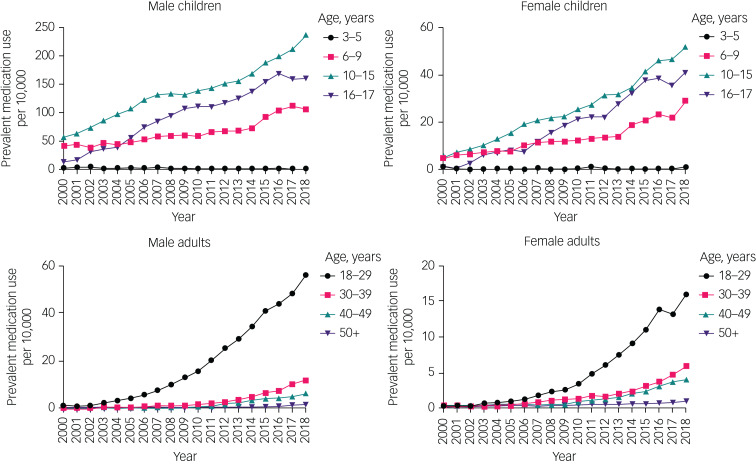
Time trends of the proportion of attention-deficit hyperactivity disorder medication prescription in children and adults, by gender and age group. Note different *y*-axis scales for each group. Raw data are given in Supplementary Table 3.

Likewise, the proportion of individuals prescribed ADHD medication increased between 2000 and 2018 in children (it quadrupled in boys and increased almost nine-fold in girls) and adults (it increased 30-fold in men and 15-fold in women) (Fig. 5, Supplementary Table 3). The proportion of individuals prescribed ADHD medication increased in all age groups, except those aged 3–5 years (Fig. 5, Supplementary Table 3).

### ADHD medication prescriptions without diagnosis

In total, 3088 individuals were prescribed at least two ADHD medications within a year during the follow-up period, but did not have an ADHD diagnostic code on their records, representing 17% of all prevalent ADHD medication users (Fig. 1). Of the 3088 individuals, 251 (8%) had a diagnosis of narcolepsy at any time in their records. Among the remainder, within 2 years of the earliest prescription, there were no clear alternative indications for the prescription.

## Discussion

We have presented data on the rate of new ADHD diagnoses in medical records and new ADHD medication prescriptions in UK primary care, along with the proportion of individuals with ADHD diagnoses and prescriptions for ADHD medication, in a large sample of almost 8 million individuals, between 2000 and 2018.

We found evidence of increasing rates of new diagnoses and prescriptions, and increases in the proportion of people with ADHD diagnoses and prescribed ADHD medications, between 2000 and 2018. Proportionately, the relative increase in the number of people diagnosed with ADHD is greatest among adults aged 18–29 years. This trend can be partially explained by a cohort effect of children with ADHD ageing into adulthood, as well as by an increased rate of new diagnoses in that age group, suggesting more ADHD diagnoses are now being made in adulthood. New ADHD medication use and the proportion of ADHD medication users have, likewise, followed this pattern.

The overall pattern of increasing rates of new diagnoses and prescriptions, and increases in the proportion of the population with ADHD diagnoses and prescriptions, is consistent with that reported internationally up to 2015,^2^ and our data suggest that this trend is continuing.

Increases in the proportion of people diagnosed with ADHD and receiving ADHD medication may have multiple drivers, which include a genuine increase in the underlying frequency of the condition; increasing awareness of the condition among patients, caregivers and medical professionals, thereby ameliorating underdiagnosis;^22^ changing attitudes toward ADHD; broadened diagnostic criteria;^23^ overdiagnosis;^24^ misdiagnosis^25^ and use of medication for ‘cognitive enhancement’ in otherwise healthy individuals.^25^ The community prevalence of ADHD (derived by sampling representative samples of the population for symptoms of ADHD) has been estimated as approximately 5% in children.^26^ If this figure applies to the UK, then even our highest prevalence estimate (2.3% in male children in 2018) suggests that only a subset of people with ADHD symptoms are identified as having ADHD in clinical practice, and that rates of clinical diagnosis of ADHD in the UK may still increase in years to come. ADHD is therefore likely underdiagnosed rather than overdiagnosed in the UK.^22^

Conversely, we found that new ADHD diagnoses and the proportion of ADHD diagnoses dropped substantially in children aged 3–5 years. Medication use remained rare in that age group throughout the study, in keeping with national guidance recommending that ADHD medication is only used for children aged <5 years in exceptional circumstances.^14^ One possibility is that clinicians are better able to correctly diagnose other neurodevelopmental disorders that can mimic ADHD in this age group. Changes in social support may have had an effect: the 1998 ‘Sure Start’ initiative increased investment in support for disadvantaged children under 5 years of age,^27^ and although many centres have been wound down, the initiative transformed early years services in local authorities and its legacy remains, including in the parenting programmes for early intervention that still run in many local authorities.^28^ In children under 5 years of age, ADHD expression often takes the form of challenging behaviour; a tendency for services to direct parents toward parenting programmes and social prescribing^29^ may have resulted in fewer children in this age group being referred to psychiatric services. The National Institute for Health and Care Excellence guidelines, which, from 2018, have recommended group-based parent training programmes as a first-line intervention for children under 5 years of age before referring to specialised ADHD services, are consistent with this approach.^14^

Further possible explanations include that general practices may have erroneously coded young children as having ADHD (perhaps intending the meaning as ‘possible ADHD’) in the past and have become more stringent in coding recently, which might reflect greater recognition of the diagnostic criteria for the condition. In addition, waiting times for specialist ADHD clinics may simply be so long that children referred when under 5 years of age are not seen until they are older.

The pattern of reduced diagnostic prevalence in children under 5 years of age was seen in one other UK primary care database study, where the prevalence of ADHD in children aged 0–5 years dropped by approximately 75% between 1998 and 2009;^30^ other previous UK-based prevalence studies reported only medication, rather than diagnostic prevalence,^2,3,5–7^ or either excluded or did not report diagnostic prevalence changes over time in this age group.^4^

The prevalence of ADHD medication usage peaked in the 10–15 year age group, and was lower in older age groups. Notably, ADHD medication usage was lower in the 16–17 year age group than the 10–15 year age group, despite a slightly higher prevalence of ADHD diagnoses. This pattern has been noted in other studies.^31,32^ This rate of decline exceeds that predicted by age-related symptom decline. Instead, this is likely to reflect significant issues in accessing transition care between child and adult services for adolescents,^33^ potentially leading to abrupt, unplanned cessation of medication.

We observed very large differences in diagnosis and prescription rates in adult age groups, with diagnostic rates in the 18–29 year age group in 2018 that were approximately three times those in the 30–39 year age group, and about twelve times greater than in the 40–49 year age group. Although changes in the community prevalence of ADHD with time could explain this, it seems more likely that there is a substantial proportion of undiagnosed ADHD in older age groups. Awareness of the diagnosis and treatment may be higher in younger adults, who are increasingly coming forward to seek ADHD care. This poses a significant challenge to health services in the UK, given that waiting times for NHS adult ADHD assessments are already long (up to 5 years).^34^

We found new ADHD diagnoses and medication usage, and the proportions of both, to be substantially higher in males compared with females in both adulthood and childhood. The male:female ratio for new diagnoses did, however, decrease in adulthood (from approximately 4:1 in childhood, reaching parity over 40 years of age), supporting prior observations that women tend to be diagnosed later in life,^35^ possibly because of under-recognition of ADHD and under-referral of girls in childhood.

We also demonstrated that the previously reported associations between ADHD incidence/prevalence and socioeconomic deprivation in UK children^4,8,9^ are retained into adulthood, for both ADHD diagnoses and prescriptions of ADHD medication. The relationship between ADHD and socioeconomic deprivation is complex. Low socioeconomic status may have a causal effect on ADHD, via increased exposure to risk factors during the pre- and perinatal period, and during childhood. There may also be genetic confounding (parents of children with ADHD having a genetic predisposition to ADHD symptoms themselves, and therefore being both more likely to suffer socioeconomic disadvantage themselves as well as pass on the genetic predisposition to ADHD to their children), labelling and reporting bias (ADHD symptoms and diagnoses potentially more likely to be reported and made in lower socioeconomic groups), and reverse causality (ADHD symptoms leading to lost income and social exclusion in both people with ADHD and their parents).^36^

Previous UK-based studies have examined diagnostic prevalence,^2,30^ medication prevalence^2,5,6^ or medication prevalence among people with ADHD diagnoses.^3,7^ We found that people prescribed ADHD medication were predominantly a subset of people with ADHD diagnoses, with only 17% of medication users having no record of a diagnosis. This proportion is considerably lower than that reported in the USA.^21^ A small proportion of medication users without ADHD diagnoses in our study had narcolepsy (8%); the remainder likely represented either uncoded ADHD or off-label use. Conversely, 42% of people with ADHD diagnoses had not received any ADHD medication from primary care. Few of these individuals may have instead received medications from secondary care only; however, a more plausible explanation is that they are not regular users of ADHD medication.

Our most recent data are from 2018. Reports suggest referral numbers for ADHD assessments, and their waiting lists, have continued to increase in the UK.^15,34^ This coincides with a surge in demand for mental healthcare during the COVID-19 pandemic, particularly for child and adolescent mental health services.^37^ It is likely that the prevalence of ADHD and ADHD medication usage has continued to increase since the end of our study period, and that both primary and secondary care have come under unprecedented pressure.

These results have clear implications for clinicians and policy makers; thus, we should be aware that ADHD may previously have been under-recognised in both children and adults. We may expect an increasing number of people needing ADHD care from both primary and specialist care, which is likely to be particularly large in areas of deprivation. Children aged 6–9 years are still the group in which ADHD is most likely to be first diagnosed, and resources should be allocated accordingly to child and adolescent psychiatry. Adult ADHD services are also in dire need of appropriate resourcing, with very long wait times in the UK,^34^ and our data suggests the need for adult ADHD care will only continue to increase. It is important that primary care clinicians are given appropriate training and support to prescribe and monitor ADHD medication safely, as ADHD medication use in primary care is increasingly common.

It has been suggested that some people with ADHD could be diagnosed and managed entirely^38^ or partially^39^ in primary care. This would be a substantial shift away from the UK's current national guidelines, which state that a diagnosis of ADHD ‘should only be made by a specialist psychiatrist, paediatrician or other appropriately qualified healthcare professional with training and expertise in the diagnosis of ADHD’.^14^ In addition, these guidelines state that ADHD should only be diagnosed based on a full clinical and psychosocial assessment, full developmental and psychiatric history and observer reports.^14^ Few primary care clinicians currently have the training to do so, or the time (a ‘straightforward’ ADHD assessment may take 2 h,^39^ whereas the standard appointment in UK primary care is 10 min long^40^). Novel solutions could involve a hybrid approach, developing the role of primary care practitioners with special expertise in ADHD in management and perhaps even diagnosis.^39^

Primary care records contain rich, longitudinal information on participants’ health. Further work could use the cohort of people with ADHD identified here to examine physical health outcomes and comorbidities in people with ADHD, which is particularly relevant given the substantial increase of adults with ADHD.

### Strengths and limitations

This study presents findings from a very large sample, using real-life primary care data, for general practices that are representative of the UK population. The findings are therefore likely generalisable within the UK.

This study only captured ADHD medication prescriptions in NHS primary care and not secondary care, which will underestimate the overall incidence and prevalence of medication usage. However, it is standard practice to transfer ongoing prescribing from both public and private secondary care sectors to NHS primary care in most cases.^14^ Given that ADHD medications are generally initiated in secondary care, and transferred to primary care once stabilised, our measure of medication use lags behind the actual inception of medication, and instead captures the point of transfer from secondary to primary care prescribing. This initiation and stabilisation period will vary, but generally takes around 8–12 weeks. This study also cannot determine the numbers of people referred to secondary care with ADHD symptoms, but rather only individuals with a confirmed diagnosis that has been communicated to primary care.

Our study period finished in 2018; since then, various events, including the COVID-19 pandemic, have had a substantial effect on mental health services. It is likely that the incidence and prevalence of ADHD in the UK has continued to change between the end of our study and the present date. Finally, as discussed above, our study captures administrative prevalence (the prevalence of coded ADHD diagnoses) and not the community prevalence of symptoms meeting the ADHD diagnostic criteria in a population-based sample.

In conclusion, the incidence and prevalence of both ADHD diagnoses and medication are highest among children, but rates have proportionally increased the most among adults between 2000 and 2018. Both adult and childhood ADHD are associated with socioeconomic deprivation. Further investment and service planning is required to ensure that the rising numbers of people with ADHD (which is likely to increase further) receive prompt, safe and effective care in both primary and secondary care.

## Data Availability

This study uses medical record data under license from IQVIA Medical Research Data (IMRD). The data supporting the findings of this study are available by application to IMRD.

## Supplementary material

For supplementary material accompanying this paper visit http://doi.org/10.1192/bjo.2023.512. click here to view supplementary material

## References

[1] Thapar A, Cooper M. Attention deficit hyperactivity disorder. Lancet 2016; 387(10024): 1240–50. 26386541 10.1016/S0140-6736(15)00238-X

[2] Raman SR, Man KKC, Bahmanyar S, Berard A, Bilder S, Boukhris T, et al. Trends in attention-deficit hyperactivity disorder medication use: a retrospective observational study using population-based databases. Lancet Psychiatry 2018; 5(10): 824–35. 30220514 10.1016/S2215-0366(18)30293-1

[3] Jick H, Kaye JA, Black C. Incidence and prevalence of drug-treated attention deficit disorder among boys in the UK. Br J Gen Pract 2004; 54(502): 345–7. 15113516 PMC1266167

[4] Hire AJ, Ashcroft DM, Springate DA, Steinke DT. ADHD in the United Kingdom: regional and socioeconomic variations in incidence rates amongst children and adolescents (2004–2013). J Atten Disord 2018; 22(2): 134–42. 26604267 10.1177/1087054715613441

[5] Beau-Lejdstrom R, Douglas I, Evans SJW, Smeeth L. Latest trends in ADHD drug prescribing patterns in children in the UK: prevalence, incidence and persistence. BMJ Open 2016; 6(6): e010508. 10.1136/bmjopen-2015-010508 PMC4932306 27297009

[6] Renoux C, Shin JY, Dell'Aniello S, Fergusson E, Suissa S. Prescribing trends of attention-deficit hyperactivity disorder (ADHD) medications in UK primary care, 1995–2015. Br J Clin Pharmacol 2016; 82(3): 858–68. 27145886 10.1111/bcp.13000 PMC5338115

[7] McCarthy S, Wilton L, Murray ML, Hodgkins P, Asherson P, Wong IC. The epidemiology of pharmacologically treated attention deficit hyperactivity disorder (ADHD) in children, adolescents and adults in UK primary care. BMC Pediatr 2012; 12: 78. 22712630 10.1186/1471-2431-12-78 PMC3472167

[8] Russell AE, Ford T, Russell G. The relationship between financial difficulty and childhood symptoms of attention deficit/hyperactivity disorder: a UK longitudinal cohort study. Soc Psychiatry Psychiatr Epidemiol 2018; 53(1): 33–44. 29124294 10.1007/s00127-017-1453-2 PMC5846873

[9] Prasad V, West J, Kendrick D, Sayal K. Attention-deficit/hyperactivity disorder: variation by socioeconomic deprivation. Arch Dis Child 2019; 104(8): 802–5. 29602777 10.1136/archdischild-2017-314470

[10] Blak BT, Thompson M, Dattani H, Bourke A. Generalisability of The Health Improvement Network (THIN) database: demographics, chronic disease prevalence and mortality rates. Inform Prim Care 2011; 19(4): 251–5. 22828580 10.14236/jhi.v19i4.820

[11] NHS Digital. Patients Registered at a GP Practice, July 2022. NHS Digital, 2022 (https://digital.nhs.uk/data-and-information/publications/statistical/patients-registered-at-a-gp-practice/july-2022).

[12] Booth N. What are the read codes? Health Libr Rev 1994; 11(3): 177–82. 10139676 10.1046/j.1365-2532.1994.1130177.x

[13] Townsend P, et al. Health and Deprivation: Inequality and the North. Croom Helm, 1988.

[14] National Institute for Health and Care Excellence (NICE). Attention Deficit Hyperactivity Disorder: Diagnosis and Management. NICE Guideline [NG87]. NICE, 2019 (https://www.nice.org.uk/guidance/ng87). 29634174

[15] Young S, Asherson P, Lloyd T, Absoud M, Arif M, Colley WA, et al. Failure of healthcare provision for attention-deficit/hyperactivity disorder in the United Kingdom: a consensus statement. Front Psychiatry 2021; 12: 649399. 10.3389/fpsyt.2021.649399 PMC8017218 33815178

[16] Maguire A, Blak BT, Thompson M. The importance of defining periods of complete mortality reporting for research using automated data from primary care. Pharmacoepidemiol Drug Saf 2009; 18(1): 76–83. 19065600 10.1002/pds.1688

[17] Horsfall L, Walters K, Petersen I. Identifying periods of acceptable computer usage in primary care research databases. Pharmacoepidemiol Drug Saf 2013; 22(1): 64–9. 23124958 10.1002/pds.3368

[18] Rocco I, Corso B, Bonati M, Minicuci N. Time of onset and/or diagnosis of ADHD in European children: a systematic review. BMC Psychiatry 2021; 21: 575. 34784913 10.1186/s12888-021-03547-x PMC8594188

[19] Lewis JD, Bilker WB, Weinstein RB, Strom BL. The relationship between time since registration and measured incidence rates in the General Practice Research Database. Pharmacoepidemiol Drug Saf 2005; 14(7): 443–51. 15898131 10.1002/pds.1115

[20] Davé S, Petersen I. Creating medical and drug code lists to identify cases in primary care databases. Pharmacoepidemiol Drug Saf 2009; 18(8): 704–7. 19455565 10.1002/pds.1770

[21] Olfson M, Blanco C, Wang S, Greenhill LL. Trends in office-based treatment of adults with stimulants in the United States. J Clin Psychiatry 2013; 74(1): 43–50. 23419225 10.4088/JCP.12m07975

[22] Taylor E. Attention deficit hyperactivity disorder: overdiagnosed or diagnoses missed? Arch Dis Child 2017; 102(4): 376–9. 27821518 10.1136/archdischild-2016-310487

[23] Batstra L, Frances A. DSM-5 further inflates attention deficit hyperactivity disorder. J Nerv Ment Dis 2012; 200(6): 486–8. 22652611 10.1097/NMD.0b013e318257c4b6

[24] Kazda L, Bell K, Thomas R, McGeechan K, Sims R, Barratt A. Overdiagnosis of attention-deficit/hyperactivity disorder in children and adolescents: a systematic scoping review. JAMA Netw Open 2021; 4(4): e215335. 33843998 10.1001/jamanetworkopen.2021.5335 PMC8042533

[25] Sibley MH. Why are stimulant medication prescriptions rising globally? Lancet Psychiatry 2018; 5(10): 774–6. 30220513 10.1016/S2215-0366(18)30317-1

[26] Polanczyk GV, Willcutt EG, Salum GA, Kieling C, Rohde LA. ADHD prevalence estimates across three decades: an updated systematic review and meta-regression analysis. Int J Epidemiol 2014; 43(2): 434–42. 24464188 10.1093/ije/dyt261 PMC4817588

[27] Melhuish E, Belsky J, Barnes J. Evaluation and value of sure start. Arch Dis Child 2010; 95(3): 159–61. 19880394 10.1136/adc.2009.161018

[28] Lindsay G, Strand S, Cullen A, Cullen S, Davis H, Conlon G, et al. Parenting Early Intervention Programme Evaluation. Department for Education, 2011 (https://www.gov.uk/government/publications/parenting-early-intervention-programme-evaluation).

[29] Hayes D, Cortina M, Labno A, Moore A, Edbrooke-Childs J, Moltrecht B, et al. Social Prescribing in Children and Young People: A Review of the Evidence. Evidence Based Practice Unit, University College London, 2020 (https://www.ucl.ac.uk/evidence-based-practice-unit/sites/evidence-based-practice-unit/files/review_social_prescribing_in_children_and_young_people_final_0.pdf).

[30] Holden SE, Jenkins-Jones S, Poole CD, Morgan CL, Coghill D, Currie CJ. The prevalence and incidence, resource use and financial costs of treating people with attention deficit/hyperactivity disorder (ADHD) in the United Kingdom (1998 to 2010). Child Adolesc Psychiatry Ment Health 2013; 7: 34. 24119376 10.1186/1753-2000-7-34 PMC3856565

[31] Newlove-Delgado T, Hamilton W, Ford TJ, Stein K, Ukoumunne OC. Prescribing for young people with attention deficit hyperactivity disorder in UK primary care: analysis of data from the Clinical Practice Research Datalink. Atten Defic Hyperact Disord 2019; 11(3): 255–62. 30730035 10.1007/s12402-019-00288-6

[32] Wong ICK, Asherson P, Bilbow A, Clifford S, Coghill D, DeSoysa R, et al. Cessation of attention deficit hyperactivity disorder drugs in the young (CADDY)–a pharmacoepidemiological and qualitative study. Health Technol Assess 2009; 13(50): iii–iv. ix–xi, 1–120. 10.3310/hta13490 19883527

[33] Eke H, Ford T, Newlove-Delgado T, Price A, Young S, Ani C, et al. Transition between child and adult services for young people with attention-deficit hyperactivity disorder (ADHD): findings from a British national surveillance study. Br J Psychiatry 2020; 217(5): 616–22. 31159893 10.1192/bjp.2019.131 PMC7589988

[34] Lindsay M. ADHD Assessment System ‘Broken’ with Five-Year Waiting Times. BBC News, 2020 (https://www.bbc.com/news/uk-england-53526174).

[35] Nussbaum NL. ADHD and female specific concerns: a review of the literature and clinical implications. J Atten Disord 2012; 16(2): 87–100. 21976033 10.1177/1087054711416909

[36] Russell G, Ford T, Rosenberg R, Kelly S. The association of attention deficit hyperactivity disorder with socioeconomic disadvantage: alternative explanations and evidence. J Child Psychol Psychiatry 2014; 55(5): 436–45. 24274762 10.1111/jcpp.12170 PMC4263245

[37] Huang HCH, Ougrin D. Impact of the COVID-19 pandemic on child and adolescent mental health services. BJPsych Open 2021; 7(5): e145. 34348823 10.1192/bjo.2021.976 PMC8353214

[38] Montano B. Diagnosis and treatment of ADHD in adults in primary care. J Clin Psychiatry 2004; 65(suppl 3): 18–21. 15046531

[39] Asherson P, Leaver L, Adamou M, Arif M, Askey G, Butler M, et al. Mainstreaming adult ADHD into primary care in the UK: guidance, practice, and best practice recommendations. BMC Psychiatry 2022; 22: 640. 36221085 10.1186/s12888-022-04290-7 PMC9553294

[40] Flaxman P. The 10-minute appointment. Br J Gen Pract 2015; 65(640): 573–4. 10.3399/bjgp15X687313 PMC4617250 26500303

