# Supplementary material for: Attention-deficit hyperactivity disorder diagnoses and prescriptions in UK primary care, 2000–2018: population-based cohort study
Source: BJPsych Open. 2023 Jul 17;9(4):e121. doi: 10.1192/bjo.2023.512 (PMC10375867; doi:10.1192/bjo.2023.512)
Supplement: Supplementary file 1 [file bjosup.zip › S2056472423005124sup001.pdf]

Year	Rate of new diagnoses, per 100,000 person years (95% confidence interval)															
	3-5		6-9		10-16		16-17		18-29		30-39		40-49		50+	
	Males	Females	Males	Females	Males	Females	Males	Females	Males	Females	Males	Females	Males	Females	Males	Females
2000	164.5 (126.12-210.88)	29.34 (14.07-53.96)	286.04 (241.38-336.57)	52.02 (32.98-78.06)	172.53 (143.5-205.71)	21.29 (11.34-36.41)	9.12 (1.1-32.95)	5.52 (0.14-30.77)	0.68 (0.02-3.79)	2.15 (0.44-6.28)	1.72 (0.35-5.02)	0 (0-2.14)	1.27 (0.15-4.6)	0 (0-2.42)	0.83 (0.17-2.42)	1.2 (0.39-2.79)
2001	146.75 (113.73-186.37)	36.06 (20.18-59.48)	305.17 (262.67-352.59)	47.14 (30.51-69.59)	135.21 (111.92-161.93)	24.21 (14.35-38.26)	3.75 (0.09-20.88)	9.03 (1.09-32.61)	2.88 (0.94-6.73)	3.06 (1-7.15)	1.43 (0.29-4.17)	1.45 (0.3-4.25)	1.05 (0.13-3.79)	1.08 (0.13-3.91)	0.46 (0.06-1.65)	1 (0.32-2.33)
2002	141.11 (111.17-176.61)	33.95 (19.78-54.36)	246.27 (211.13-285.58)	50.38 (34.46-71.12)	162.24 (138.77-188.55)	25.58 (16.21-38.38)	15.43 (5.01-36.02)	11.07 (2.28-32.36)	3.38 (1.36-6.97)	0.51 (0.01-2.86)	1.22 (0.25-3.55)	1.65 (0.45-4.23)	0.87 (0.11-3.15)	0.45 (0.01-2.51)	0.39 (0.05-1.39)	1.01 (0.37-2.2)
2003	109.49 (84.5-139.55)	12.52 (5.03-25.8)	344.7 (305.09-388.03)	59.81 (43.28-80.56)	156.83 (135.04-181.13)	23.57 (15.1-35.07)	40.57 (22.7-66.91)	0 (0-11.84)	8.19 (4.93-12.79)	2.29 (0.74-5.34)	3.32 (1.52-6.3)	0.75 (0.09-2.72)	0.39 (0.01-2.15)	1.2 (0.25-3.5)	0.52 (0.11-1.51)	1.06 (0.43-2.19)
2004	132.36 (105.72-163.66)	22.97 (12.56-38.54)	294.45 (259.61-332.65)	62.31 (46.25-82.14)	198.86 (175.37-224.61)	44.12 (32.75-58.17)	36.03 (20.16-59.42)	8.48 (1.75-24.79)	7.41 (4.46-11.58)	2.5 (0.92-5.44)	0.69 (0.08-2.48)	1.75 (0.57-4.1)	0.69 (0.08-2.5)	2.15 (0.79-4.68)	0.94 (0.34-2.05)	0.14 (0-0.77)
2005	121.33 (96.2-151)	33.28 (20.6-50.88)	276.62 (243.7-312.73)	80.91 (62.95-102.4)	206.34 (182.96-231.88)	35.81 (25.92-48.24)	27.05 (13.98-47.26)	7.93 (1.63-23.16)	7 (4.21-10.93)	3.14 (1.36-6.19)	4.02 (2.08-7.02)	2.75 (1.19-5.41)	2.59 (1.12-5.1)	2.34 (0.94-4.83)	1.33 (0.61-2.53)	1.57 (0.81-2.75)
2006	102.37 (79.65-129.56)	26.32 (15.33-42.13)	324.73 (289.36-363.23)	69.14 (52.88-88.81)	220.81 (196.87-246.86)	42.64 (31.94-55.78)	58.56 (38.59-85.19)	15.18 (5.57-33.04)	5.67 (3.24-9.21)	3.39 (1.55-6.44)	4.7 (2.57-7.89)	1.03 (0.21-3.02)	1.55 (0.5-3.62)	1.6 (0.52-3.74)	0.43 (0.09-1.25)	0.76 (0.28-1.66)
2007	104.88 (82.21-131.87)	22.55 (12.62-37.2)	350.71 (314.16-390.36)	59.68 (44.83-77.87)	208.29 (185.31-233.33)	40.51 (30.25-53.12)	41.71 (25.48-64.42)	17.01 (6.84-35.05)	5.78 (3.37-9.26)	2.52 (1.01-5.2)	4.38 (2.33-7.49)	4.5 (2.39-7.69)	2.1 (0.84-4.32)	2.48 (1.07-4.88)	2.63 (1.58-4.11)	1.98 (1.13-3.21)
2008	94.71 (73.69-119.87)	26 (15.41-41.08)	342.48 (306.42-381.62)	77.52 (60.54-97.78)	199.33 (177.08-223.6)	50.51 (39.15-64.15)	28.49 (15.58-47.8)	9.47 (2.58-24.26)	7.59 (4.81-11.39)	6.25 (3.7-9.88)	2.72 (1.17-5.36)	4.17 (2.16-7.29)	3.52 (1.82-6.14)	3.32 (1.66-5.94)	1.35 (0.65-2.48)	1.33 (0.66-2.38)
2009	94.12 (73.37-118.91)	18.34 (9.77-31.36)	329.31 (293.94-367.76)	66.33 (50.74-85.2)	202.17 (179.86-226.47)	41.29 (31.19-53.62)	73.68 (51.61-102.01)	30.62 (16.3-52.36)	12.62 (8.98-17.26)	6.46 (3.89-10.08)	5.47 (3.13-8.89)	5.25 (2.94-8.66)	3.19 (1.59-5.7)	3.58 (1.85-6.25)	1.73 (0.92-2.95)	1.55 (0.83-2.65)
2010	106.01 (83.79-132.3)	18.48 (9.84-31.6)	386.75 (347.99-428.66)	79.91 (62.52-100.63)	193.71 (171.6-217.87)	44.93 (34.29-57.83)	65.7 (44.64-93.25)	21.72 (9.93-41.24)	11.94 (8.36-16.53)	4.86 (2.66-8.16)	6.4 (3.79-10.11)	5.81 (3.32-9.43)	2.97 (1.42-5.46)	1.83 (0.67-3.98)	1.35 (0.65-2.47)	1.21 (0.58-2.23)
2011	86.21 (66.53-109.88)	18.01 (9.59-30.79)	392.34 (353.68-434.08)	93.44 (74.74-115.4)	172.28 (151.55-195.07)	58.03 (45.94-72.33)	50.43 (32.31-75.04)	18.9 (8.16-37.24)	17.1 (12.77-22.43)	7.88 (5-11.82)	7.52 (4.65-11.49)	6.54 (3.87-10.33)	3.86 (2.05-6.6)	5.45 (3.23-8.61)	2.24 (1.31-3.59)	1.79 (1-2.95)
2012	79.17 (60.56-101.7)	19.08 (10.43-32.01)	436.12 (395.68-479.57)	100.96 (81.69-123.42)	226.46 (202.58-252.39)	66.32 (53.39-81.42)	75.6 (52.95-104.66)	30.03 (15.99-51.35)	20.26 (15.53-25.97)	9.14 (6.02-13.3)	12.64 (8.8-17.58)	8.04 (5.04-12.17)	4.78 (2.73-7.76)	3.03 (1.46-5.58)	2.22 (1.29-3.55)	2.12 (1.26-3.35)
2013	90.5 (70.28-114.73)	18.21 (9.7-31.14)	474.88 (432.24-520.58)	122.62 (101.06-147.43)	218.9 (194.95-244.99)	52.75 (41.12-66.64)	78.02 (54.64-108.01)	28.37 (14.66-49.56)	22.51 (17.41-28.64)	14 (10-19.06)	11.72 (7.96-16.63)	7.24 (4.36-11.3)	5.67 (3.36-8.96)	5.75 (3.41-9.09)	3.49 (2.28-5.11)	4.26 (2.97-5.92)
2014	72.07 (53.66-94.76)	23.85 (13.63-38.73)	474.68 (431.11-521.45)	108.66 (88.02-132.7)	228.24 (202.98-255.78)	60.59 (47.73-75.83)	95.58 (68.88-129.19)	34.68 (18.96-58.19)	34.14 (27.59-41.78)	19.27 (14.39-25.27)	20.98 (15.67-27.52)	13.83 (9.58-19.33)	10.59 (7.2-15.03)	8.66 (5.6-12.78)	4.77 (3.3-6.67)	5.62 (4.08-7.54)
2015	110.49 (85.63-140.32)	15.6 (7.13-29.62)	563 (511.96-617.76)	154.39 (127.8-184.9)	230.12 (202.74-260.16)	76.73 (61.11-95.11)	74.62 (49.59-107.85)	25.75 (11.77-48.87)	34.64 (27.51-43.06)	16.52 (11.69-22.67)	22.15 (16.28-29.46)	13.29 (8.83-19.21)	11.09 (7.31-16.13)	6.65 (3.8-10.79)	3.42 (2.12-5.23)	4.91 (3.38-6.89)
2016	119.22 (90.76-153.79)	12.78 (4.69-27.81)	684.11 (622.62-750.03)	183.63 (151.87-220.08)	271.05 (238.36-306.97)	107.78 (87.4-131.48)	82.02 (53.08-121.07)	48.31 (26.41-81.06)	37.51 (29.35-47.24)	15.87 (10.71-22.66)	23.76 (17.13-32.12)	14.8 (9.67-21.68)	10.26 (6.27-15.84)	13.49 (8.81-19.77)	6.91 (4.81-9.61)	8.66 (6.38-11.48)
2017	121.89 (90.18-161.15)	20.9 (9.02-41.18)	797.82 (725.56-875.32)	194.22 (158.85-235.11)	373.45 (331.77-418.91)	89.9 (69.95-113.77)	148.41 (105.02-203.71)	68.92 (40.15-110.34)	46.56 (36.56-58.45)	25.3 (17.99-34.58)	29.4 (21.36-39.47)	13.48 (8.23-20.82)	13.62 (8.53-20.61)	11.93 (7.18-18.63)	6.01 (3.93-8.81)	5.73 (3.78-8.34)
2018	85.65 (56.91-123.78)	19.35 (7.1-42.13)	746.96 (671.68-828.36)	230.93 (189.25-279.05)	363.09 (319.56-410.9)	99.96 (77.62-126.72)	81.67 (48.4-129.08)	64.58 (35.31-108.36)	50 (38.75-63.5)	39.45 (29.28-52.01)	33.82 (24.47-45.55)	24.66 (16.75-35)	19.63 (12.93-28.55)	13.24 (7.85-20.93)	6.95 (4.58-10.12)	5.93 (3.84-8.76)

Year	Rate of new prescriptions, per 100,000 person years (95% confidence interval)															
	3-5		6-9		10-16		16-17		18-29		30-39		40-49		50+	
	Males	Females	Males	Females	Males	Females	Males	Females	Males	Females	Males	Females	Males	Females	Males	Females
2000	26.44 (12.68-48.62)	17.59 (6.45-38.28)	233.06 (193.07-278.89)	38.38 (22.36-61.44)	135.57 (110.06-165.21)	19.63 (10.14-34.28)	4.55 (0.12-25.33)	0 (0-20.36)	3.4 (1.1-7.93)	0.72 (0.02-3.99)	1.72 (0.35-5.02)	1.16 (0.14-4.19)	1.27 (0.15-4.6)	1.97 (0.41-5.76)	1.38 (0.45-3.22)	0.96 (0.26-2.45)
2001	56.79 (37.09-83.21)	2.4 (0.06-13.39)	212.51 (177.43-252.51)	30.11 (17.21-48.9)	151.52 (126.86-179.57)	25.52 (15.36-39.85)	18.67 (6.06-43.56)	0 (0-16.63)	0 (0-2.12)	1.23 (0.15-4.43)	0.48 (0.01-2.65)	1.45 (0.3-4.25)	1.05 (0.13-3.79)	0 (0-2)	1.37 (0.5-2.99)	1.4 (0.56-2.88)
2002	42.59 (27-63.91)	2 (0.05-11.12)	188.74 (158.25-223.4)	37.72 (24.17-56.12)	160.31 (137.05-186.38)	23.32 (14.43-35.64)	27.67 (12.65-52.53)	14.75 (4.02-37.76)	2.41 (0.78-5.63)	1.03 (0.12-3.71)	2.02 (0.66-4.73)	0.83 (0.1-2.98)	0.87 (0.11-3.15)	0.45 (0.01-2.51)	0.77 (0.21-1.97)	1.18 (0.47-2.43)
2003	40.34 (25.85-60.03)	7.15 (1.95-18.31)	238.75 (206.09-275.11)	34.71 (22.47-51.25)	169.26 (146.67-194.34)	17.65 (10.46-27.89)	32.3 (16.69-56.42)	6.41 (0.78-23.16)	3.01 (1.21-6.21)	3.2 (1.29-6.6)	2.58 (1.04-5.32)	0.38 (0.01-2.1)	1.16 (0.24-3.39)	1.2 (0.25-3.5)	0.86 (0.28-2.02)	1.67 (0.83-2.99)
2004	52.86 (36.6-73.86)	8.2 (2.66-19.14)	243.83 (212.32-278.69)	49.77 (35.55-67.77)	198.16 (174.8-223.77)	39.63 (28.9-53.03)	28.66 (14.81-50.06)	5.65 (0.68-20.41)	3.89 (1.87-7.16)	2.08 (0.68-4.86)	0 (0-1.27)	1.75 (0.57-4.09)	2.08 (0.76-4.53)	0.72 (0.09-2.59)	1.57 (0.75-2.88)	0.97 (0.39-2)
2005	40.89 (26.95-59.5)	12.68 (5.47-24.98)	251.3 (220.07-285.73)	52.69 (38.43-70.51)	208.33 (184.92-233.88)	40.72 (30.13-53.84)	42.54 (25.61-66.43)	2.64 (0.07-14.7)	6.61 (3.92-10.45)	2.75 (1.1-5.66)	2.34 (0.94-4.83)	1.03 (0.21-3.01)	1.3 (0.35-3.32)	1 (0.21-2.94)	1.78 (0.92-3.1)	1.57 (0.81-2.75)
2006	29.64 (18.1-45.77)	9.29 (3.41-20.21)	286.15 (253.09-322.32)	58.86 (43.96-77.19)	252.38 (226.84-280.01)	46.57 (35.37-60.21)	36.58 (21.31-58.57)	10.1 (2.75-25.86)	7.07 (4.32-10.92)	2.64 (1.06-5.43)	3.69 (1.84-6.61)	2.41 (0.97-4.97)	1.86 (0.68-4.05)	1.92 (0.71-4.19)	0.86 (0.32-1.87)	1.53 (0.79-2.67)
2007	54.53 (38.59-74.85)	15.03 (7.21-27.64)	310.56 (276.3-347.9)	61.82 (46.7-80.28)	225.43 (201.58-251.32)	42.77 (32.22-55.67)	41.36 (25.27-63.88)	14.55 (5.34-31.67)	6.44 (3.88-10.06)	2.16 (0.79-4.71)	2.69 (1.16-5.31)	3.11 (1.42-5.91)	1.8 (0.66-3.91)	2.17 (0.87-4.46)	1.66 (0.86-2.9)	1.24 (0.59-2.27)
2008	41.14 (27.76-58.73)	2.89 (0.35-10.43)	307.8 (273.73-344.93)	61.08 (46.14-79.32)	189.64 (168.04-213.25)	54.18 (42.39-68.23)	32.29 (18.46-52.44)	16.55 (6.65-34.1)	13.47 (9.67-18.27)	4.16 (2.15-7.27)	3.74 (1.86-6.68)	2.43 (0.98-5.01)	2.34 (1.01-4.62)	1.51 (0.49-3.52)	2.16 (1.23-3.5)	0.6 (0.2-1.41)
2009	32.24 (20.66-47.98)	8.46 (3.11-18.42)	295.81 (262.4-332.29)	58.66 (44.07-76.54)	181.12 (160.12-204.11)	47.84 (36.92-60.98)	50.71 (32.82-74.86)	18.8 (8.12-37.05)	9.66 (6.52-13.79)	3.39 (1.63-6.24)	4.1 (2.12-7.17)	4.55 (2.42-7.78)	1.74 (0.64-3.78)	1.19 (0.33-3.05)	1.86 (1.02-3.12)	1.19 (0.57-2.2)
2010	29.88 (18.72-45.23)	4.26 (0.88-12.46)	330.18 (294.5-368.99)	63.2 (47.87-81.89)	208.98 (186.07-233.93)	51.59 (40.14-65.28)	56.66 (37.34-82.43)	26.49 (13.22-47.4)	9.89 (6.67-14.12)	3.81 (1.9-6.83)	3.91 (1.95-6.99)	6.17 (3.59-9.87)	2.67 (1.22-5.07)	1.52 (0.5-3.56)	1.35 (0.65-2.48)	1.09 (0.5-2.07)
2011	27.83 (17.23-42.55)	8.31 (3.05-18.09)	358.83 (321.96-398.76)	74.91 (58.28-94.8)	182.9 (161.58-206.25)	46.95 (36.16-59.96)	66.62 (45.57-94.05)	14.14 (5.19-30.79)	16.66 (12.41-21.91)	8.9 (5.81-13.03)	6.08 (3.54-9.74)	5.81 (3.32-9.44)	2.67 (1.22-5.07)	2.12 (0.85-4.36)	1.58 (0.82-2.77)	1.07 (0.49-2.04)
2012	33.73 (22.03-49.42)	9.54 (3.84-19.65)	359.28 (322.72-398.84)	71.15 (55.14-90.36)	226.54 (202.72-252.39)	61.87 (49.42-76.5)	64.56 (43.87-91.64)	23.06 (11.06-42.4)	25.3 (20-31.57)	11.16 (7.68-15.67)	12.27 (8.5-17.14)	4.38 (2.27-7.66)	6.57 (4.12-9.94)	3.64 (1.88-6.36)	1.83 (1-3.06)	1.06 (0.49-2.02)
2013	30.6 (19.4-45.91)	2.8 (0.34-10.12)	349.43 (313.06-388.86)	99.75 (80.41-122.34)	218.41 (194.54-244.39)	46.66 (35.78-59.82)	73.09 (50.62-102.14)	28.32 (14.63-49.47)	25.38 (19.96-31.81)	9.43 (6.22-13.73)	11.33 (7.64-16.17)	6.09 (3.48-9.89)	5.35 (3.12-8.57)	3.51 (1.75-6.29)	1.48 (0.74-2.64)	1.22 (0.58-2.24)
2014	25.42 (15.07-40.18)	5.96 (1.62-15.26)	417.42 (376.7-461.33)	111.97 (91-136.32)	230.82 (205.48-258.43)	48.57 (37.15-62.39)	83.54 (58.82-115.15)	22.26 (10.18-42.25)	29.52 (23.48-36.64)	16.28 (11.83-21.85)	18.13 (13.22-24.26)	6.91 (4.03-11.07)	6.49 (3.91-10.13)	4.85 (2.65-8.13)	0.56 (0.15-1.44)	0.77 (0.28-1.67)
2015	57.69 (40.18-80.23)	3.47 (0.42-12.52)	489.23 (441.82-540.35)	118.97 (95.78-146.06)	276.51 (246.49-309.18)	83.1 (66.82-102.14)	111.09 (80.07-150.17)	42.84 (23.98-70.66)	37.7 (30.28-46.4)	14.32 (9.85-20.1)	22.11 (16.25-29.4)	9.49 (5.8-14.66)	8.62 (5.34-13.18)	4.98 (2.58-8.71)	1.96 (1.01-3.42)	1.49 (0.71-2.74)
2016	36.35 (21.54-57.45)	4.26 (0.52-15.39)	564.3 (508.69-624.32)	117.55 (92.46-147.35)	253.29 (221.81-287.99)	77.67 (60.55-98.14)	65.17 (39.81-100.65)	51.68 (28.92-85.24)	33.52 (25.87-42.73)	18.48 (12.87-25.7)	16.93 (11.42-24.17)	10.24 (6.07-16.18)	6.67 (3.55-11.4)	9.86 (5.94-15.4)	2.76 (1.51-4.64)	2.35 (1.25-4.01)
2017	19.89 (8.59-39.18)	5.22 (0.63-18.87)	571.84 (511.08-637.83)	105.23 (79.7-136.34)	338 (298.53-381.25)	76.74 (58.42-98.99)	104.82 (69.08-152.51)	32.39 (13.98-63.82)	48.58 (38.4-60.63)	16.18 (10.47-23.89)	26.65 (19.04-36.29)	11.45 (6.67-18.33)	10.52 (6.13-16.84)	5.02 (2.17-9.9)	3.7 (2.11-6.01)	1.91 (0.87-3.63)
2018	36.69 (18.96-64.08)	6.45 (0.78-23.31)	543.89 (480.14-613.76)	165.86 (130.89-207.3)	329.2 (287.94-374.71)	96.81 (74.87-123.17)	121.81 (80.27-177.23)	55.26 (28.56-96.53)	47.3 (36.43-60.4)	40.14 (29.89-52.78)	28.2 (19.75-39.04)	23.85 (16.09-34.04)	15.26 (9.44-23.32)	11.03 (6.17-18.2)	2.58 (1.24-4.74)	1.66 (0.67-3.42)
