# Supplementary material for: Attention-deficit hyperactivity disorder diagnoses and prescriptions in UK primary care, 2000–2018: population-based cohort study
Source: BJPsych Open. 2023 Jul 17;9(4):e121. doi: 10.1192/bjo.2023.512 (PMC10375867; doi:10.1192/bjo.2023.512)
Supplement: Supplementary file 1 [file bjosup.zip › S2056472423005124sup002.pdf]

Year	Proportion with ADHD diagnosis, per 10,000 (95% confidence interval)															
	3-5		6-9		10-16		16-17		18-29		30-39		40-49		50+	
	Males	Females	Males	Females	Males	Females	Males	Females	Males	Females	Males	Females	Males	Females	Males	Females
2000	43.1 (34.28-53.49)	12.24 (7.58-18.71)	130.1 (119-141.96)	31.59 (25.87-38.21)	138.52 (128.97-148.58)	23.54 (19.37-28.35)	76.78 (64.2-91.11)	10.04 (5.49-16.84)	14.41 (12.22-16.89)	3.06 (2.07-4.37)	5.36 (4.18-6.78)	2.01 (1.31-2.94)	1.64 (1-2.53)	2.11 (1.37-3.11)	1.02 (0.68-1.47)	2.06 (1.59-2.61)
2001	42.57 (34.99-51.31)	9 (5.57-13.75)	130.52 (120.81-140.79)	30.14 (25.27-35.67)	154.54 (145.8-163.66)	29.28 (25.22-33.8)	91.61 (79.64-104.87)	14.21 (9.36-20.67)	16.6 (14.54-18.87)	3.42 (2.48-4.59)	5.27 (4.24-6.46)	1.94 (1.33-2.72)	2.04 (1.4-2.87)	2.16 (1.5-3.02)	0.96 (0.67-1.33)	1.93 (1.54-2.4)
2002	37.32 (30.87-44.73)	8.25 (5.29-12.28)	117.23 (108.88-126.06)	26.28 (22.17-30.92)	169.92 (161.64-178.53)	31.88 (28.05-36.08)	107.52 (95.81-120.27)	17.28 (12.4-23.44)	22.77 (20.56-25.16)	4.13 (3.18-5.27)	4.85 (3.95-5.89)	1.91 (1.36-2.61)	2.6 (1.94-3.4)	1.7 (1.17-2.38)	0.79 (0.55-1.09)	1.86 (1.51-2.27)
2003	27.16 (22.02-33.13)	8.03 (5.29-11.68)	121.28 (113.36-129.62)	25.54 (21.79-29.75)	186.4 (178.33-194.75)	33.75 (30.09-37.73)	127.54 (115.77-140.19)	22.08 (16.93-28.3)	29.83 (27.48-32.33)	5.25 (4.25-6.42)	5.47 (4.57-6.5)	2.21 (1.64-2.9)	2.69 (2.07-3.44)	1.71 (1.22-2.34)	0.74 (0.52-1.01)	1.65 (1.34-2.01)
2004	22.7 (18.18-28)	5.74 (3.55-8.77)	114.93 (107.63-122.6)	24.7 (21.23-28.57)	205.99 (197.96-214.26)	38.71 (35.01-42.7)	157.68 (145.41-170.7)	25.8 (20.55-31.98)	38 (35.5-40.62)	6.3 (5.26-7.48)	5.43 (4.56-6.41)	2.4 (1.83-3.09)	2.65 (2.06-3.35)	1.87 (1.38-2.48)	0.92 (0.69-1.2)	1.64 (1.35-1.98)
2005	20.84 (16.71-25.67)	3.97 (2.27-6.45)	111.05 (104.21-118.22)	25.04 (21.72-28.72)	220.76 (212.84-228.9)	41.19 (37.57-45.07)	194.47 (181.52-208.09)	34.05 (28.3-40.63)	48.98 (46.28-51.79)	8.13 (7-9.39)	5.86 (4.98-6.84)	2.84 (2.24-3.56)	3.38 (2.75-4.11)	2.3 (1.78-2.93)	1.02 (0.79-1.29)	1.75 (1.46-2.08)
2006	18.59 (14.74-23.14)	4.61 (2.78-7.2)	110.78 (104.04-117.85)	23.97 (20.78-27.5)	236.38 (228.32-244.64)	46.11 (42.36-50.12)	220.74 (207.3-234.81)	37.67 (31.77-44.35)	62.6 (59.63-65.69)	10.39 (9.15-11.76)	6.34 (5.44-7.36)	3.12 (2.49-3.87)	3.8 (3.14-4.55)	2.28 (1.78-2.89)	1.13 (0.89-1.41)	1.7 (1.42-2.02)
2007	17.33 (13.68-21.66)	4.44 (2.67-6.93)	108.3 (101.63-115.29)	23.56 (20.43-27.05)	243.51 (235.4-251.84)	45.81 (42.11-49.75)	246.25 (232.29-260.83)	41.29 (35.19-48.13)	78.75 (75.47-82.13)	13.63 (12.23-15.16)	7.65 (6.64-8.76)	3.77 (3.07-4.59)	4.09 (3.41-4.85)	2.42 (1.91-3.04)	1.39 (1.13-1.69)	1.83 (1.55-2.16)
2008	15.15 (11.83-19.11)	3.63 (2.08-5.9)	106.64 (100.07-113.52)	23.02 (19.96-26.43)	249.44 (241.39-257.7)	49.55 (45.79-53.53)	258.59 (244.5-273.28)	46.04 (39.7-53.12)	97.2 (93.64-100.87)	17 (15.47-18.65)	8.32 (7.28-9.47)	4.27 (3.52-5.12)	4.47 (3.78-5.25)	2.82 (2.27-3.47)	1.56 (1.29-1.88)	1.82 (1.54-2.14)
2009	9.31 (6.79-12.46)	3.03 (1.66-5.08)	105.65 (99.08-112.54)	22.18 (19.16-25.54)	246.27 (238.23-254.52)	48.34 (44.64-52.27)	289.15 (274.04-304.87)	55.45 (48.43-63.21)	116.54 (112.62-120.56)	20.95 (19.24-22.78)	9.14 (8.03-10.35)	4.69 (3.9-5.6)	5.21 (4.46-6.05)	3.24 (2.65-3.93)	1.87 (1.57-2.21)	1.96 (1.66-2.29)
2010	11.64 (8.8-15.12)	3.65 (2.13-5.84)	103.02 (96.54-109.81)	22.17 (19.15-25.54)	244.33 (236.27-252.59)	48.8 (45.08-52.74)	312.3 (296.55-328.67)	65.31 (57.66-73.7)	137.3 (133.04-141.67)	24.42 (22.57-26.38)	11.09 (9.86-12.44)	5.56 (4.69-6.55)	5.97 (5.15-6.87)	3.69 (3.05-4.42)	2.02 (1.7-2.37)	2.15 (1.85-2.5)
2011	14.61 (11.43-18.39)	4.46 (2.76-6.82)	106.24 (99.66-113.14)	25.14 (21.92-28.7)	245.15 (237.01-253.49)	50.44 (46.64-54.45)	294.59 (279.18-310.63)	64.26 (56.68-72.56)	162.52 (157.86-167.28)	29.83 (27.78-32)	14.59 (13.14-16.15)	6.3 (5.36-7.36)	6.48 (5.62-7.43)	4.36 (3.65-5.15)	2.44 (2.09-2.82)	2.37 (2.05-2.73)
2012	11.82 (8.98-15.28)	2.79 (1.49-4.78)	110.89 (104.27-117.82)	25.45 (22.26-28.97)	249.29 (241.11-257.67)	54.93 (51-59.08)	289 (273.82-304.8)	63.48 (56.08-71.58)	184.91 (179.99-189.94)	35 (32.8-37.31)	17.32 (15.75-19.01)	7.48 (6.45-8.62)	7.75 (6.82-8.79)	4.62 (3.9-5.43)	2.73 (2.37-3.13)	2.59 (2.25-2.96)
2013	13.74 (10.63-17.49)	2.85 (1.52-4.87)	116.59 (109.65-123.85)	28.13 (24.7-31.91)	252.83 (244.32-261.57)	55.51 (51.44-59.81)	310.13 (294.04-326.87)	64.87 (57.2-73.29)	204.94 (199.57-210.41)	40.55 (38.1-43.1)	23.29 (21.4-25.31)	8.12 (7.02-9.35)	8.87 (7.83-10.02)	5.34 (4.54-6.25)	3.11 (2.71-3.55)	3.05 (2.67-3.46)
2014	9.53 (6.87-12.88)	4.31 (2.55-6.81)	121.83 (114.48-129.53)	30.11 (26.43-34.15)	264.6 (255.5-273.94)	59.84 (55.43-64.49)	310.45 (293.71-327.9)	72.18 (63.8-81.36)	230.72 (224.78-236.78)	46.96 (44.22-49.83)	30.77 (28.49-33.19)	10.06 (8.76-11.48)	10.76 (9.54-12.09)	6.67 (5.72-7.74)	3.45 (3.02-3.93)	3.6 (3.18-4.07)
2015	14.08 (10.48-18.51)	3.5 (1.81-6.12)	141.38 (132.7-150.47)	38.02 (33.47-43.01)	282.9 (272.46-293.63)	67.4 (62.26-72.86)	321.63 (302.71-341.42)	82.53 (72.68-93.36)	257.23 (250.28-264.32)	53.33 (50.1-56.71)	39.8 (36.94-42.82)	11.75 (10.22-13.46)	12.5 (11.03-14.12)	8.17 (6.98-9.51)	4.1 (3.58-4.68)	3.72 (3.25-4.25)
2016	18.1 (13.64-23.56)	2.79 (1.2-5.49)	160.68 (150.84-170.98)	44.66 (39.42-50.41)	296.11 (284.73-307.84)	74.97 (69.18-81.11)	344.12 (323.05-366.21)	82.19 (71.73-93.74)	274.8 (267.11-282.65)	59.18 (55.53-63)	50.67 (47.25-54.28)	14.16 (12.37-16.14)	13.69 (12.01-15.53)	9.65 (8.24-11.22)	4.65 (4.06-5.31)	4.48 (3.92-5.1)
2017	16.68 (12.02-22.55)	1.25 (0.26-3.64)	183.7 (172.22-195.74)	47.44 (41.56-53.93)	319.28 (306.51-332.45)	82.49 (75.94-89.46)	314.7 (292.71-337.91)	81.77 (70.47-94.36)	293.86 (285.14-302.79)	64.41 (60.21-68.83)	63.41 (59.26-67.78)	18.27 (16.05-20.7)	15.81 (13.84-17.98)	11.22 (9.56-13.08)	5.38 (4.69-6.15)	4.96 (4.32-5.66)
2018	9 (5.42-14.06)	1.5 (0.31-4.38)	189.35 (176.94-202.39)	52.71 (46.12-59.98)	353.85 (339.84-368.28)	90.61 (83.43-98.24)	323.95 (300.19-349.09)	91.03 (78.48-105.02)	318.4 (308.69-328.34)	75.47 (70.54-80.65)	84.58 (79.43-89.97)	22.8 (20.14-25.71)	18.87 (16.56-21.4)	12.59 (10.72-14.7)	6.39 (5.6-7.25)	5.64 (4.93-6.42)

Year	Proportion with current ADHD medication use, per 10,000 (95% confidence interval)															
	3-5		6-9		10-16		16-17		18-29		30-39		40-49		50+	
	Males	Females	Males	Females	Males	Females	Males	Females	Males	Females	Males	Females	Males	Females	Males	Females
2000	2.1 (0.57-5.38)	1.17 (0.14-4.21)	41.22 (35.08-48.13)	4.77 (2.73-7.74)	56.93 (50.87-63.51)	5.3 (3.43-7.83)	13.48 (8.55-20.23)	0 (0-2.65)	1.13 (0.58-1.97)	0.1 (0-0.57)	0.23 (0.05-0.67)	0.39 (0.13-0.9)	0.33 (0.09-0.84)	0.34 (0.09-0.86)	0.25 (0.1-0.51)	0.31 (0.15-0.56)
2001	3.47 (1.59-6.59)	0.43 (0.01-2.38)	44.33 (38.76-50.47)	6.01 (3.96-8.74)	63.85 (58.29-69.8)	7.06 (5.15-9.45)	16.52 (11.69-22.68)	0 (0-1.94)	0.92 (0.49-1.57)	0.23 (0.05-0.68)	0.17 (0.04-0.51)	0.41 (0.16-0.84)	0.43 (0.17-0.89)	0.32 (0.1-0.74)	0.16 (0.06-0.35)	0.23 (0.11-0.43)
2002	4.45 (2.43-7.47)	0 (0-1.26)	38.32 (33.62-43.5)	6.5 (4.55-9)	73.35 (67.95-79.07)	8.52 (6.6-10.82)	31.53 (25.36-38.76)	2.52 (0.93-5.49)	1.12 (0.67-1.75)	0.19 (0.04-0.56)	0.33 (0.13-0.69)	0.24 (0.08-0.57)	0.45 (0.2-0.85)	0.36 (0.14-0.74)	0.17 (0.08-0.34)	0.25 (0.13-0.43)
2003	1.95 (0.79-4.03)	0.3 (0.01-1.65)	46.58 (41.72-51.84)	7.25 (5.33-9.65)	85.06 (79.64-90.74)	10.24 (8.28-12.53)	35.52 (29.45-42.48)	6.04 (3.52-9.67)	2.2 (1.6-2.95)	0.66 (0.34-1.16)	0.38 (0.17-0.72)	0.17 (0.05-0.44)	0.43 (0.2-0.78)	0.44 (0.21-0.81)	0.19 (0.09-0.35)	0.25 (0.14-0.41)
2004	2.86 (1.43-5.12)	0.27 (0.01-1.52)	44.27 (39.79-49.12)	7.76 (5.88-10.05)	97.39 (91.9-103.13)	12.88 (10.78-15.26)	38.96 (32.99-45.69)	7.14 (4.52-10.71)	3.13 (2.45-3.95)	0.73 (0.41-1.2)	0.31 (0.13-0.61)	0.24 (0.09-0.52)	0.26 (0.11-0.54)	0.58 (0.33-0.96)	0.27 (0.16-0.44)	0.27 (0.16-0.43)
2005	3.31 (1.81-5.55)	0.5 (0.06-1.79)	47.43 (43.01-52.2)	7.71 (5.93-9.87)	106.52 (101.04-112.22)	15.35 (13.17-17.79)	55.82 (48.99-63.33)	8.01 (5.37-11.51)	4.21 (3.45-5.1)	0.96 (0.6-1.45)	0.51 (0.28-0.86)	0.34 (0.15-0.64)	0.3 (0.14-0.58)	0.52 (0.29-0.86)	0.26 (0.15-0.42)	0.33 (0.21-0.49)
2006	2.32 (1.11-4.27)	0.24 (0.01-1.35)	52.74 (48.13-57.68)	10.25 (8.21-12.64)	122.6 (116.82-128.59)	19.24 (16.84-21.88)	74.04 (66.35-82.37)	7.31 (4.86-10.57)	5.67 (4.8-6.65)	1.28 (0.87-1.81)	0.76 (0.47-1.16)	0.59 (0.34-0.96)	0.26 (0.11-0.51)	0.5 (0.28-0.82)	0.34 (0.21-0.51)	0.35 (0.23-0.51)
2007	3.37 (1.89-5.56)	0.7 (0.14-2.04)	57.77 (52.93-62.93)	11.41 (9.26-13.9)	131.53 (125.59-137.68)	20.6 (18.15-23.29)	85.24 (77.12-93.99)	11.63 (8.51-15.51)	7.6 (6.61-8.7)	1.79 (1.3-2.39)	1.06 (0.71-1.53)	0.83 (0.52-1.25)	0.44 (0.24-0.74)	0.45 (0.25-0.76)	0.37 (0.24-0.54)	0.31 (0.2-0.46)
2008	1.49 (0.6-3.07)	0 (0-0.83)	59.32 (54.46-64.5)	11.72 (9.56-14.21)	133.45 (127.58-139.52)	21.76 (19.3-24.45)	94.91 (86.46-103.96)	15.4 (11.83-19.7)	10.07 (8.95-11.3)	2.3 (1.76-2.95)	1.09 (0.73-1.55)	1.04 (0.69-1.5)	0.36 (0.19-0.63)	0.46 (0.26-0.77)	0.4 (0.27-0.57)	0.31 (0.2-0.45)
2009	1.86 (0.85-3.53)	0 (0-0.8)	60.11 (55.18-65.35)	11.93 (9.75-14.45)	131.77 (125.91-137.83)	22.36 (19.87-25.07)	107.22 (98.11-116.95)	18.53 (14.58-23.23)	13.03 (11.75-14.42)	2.57 (2-3.26)	1.19 (0.81-1.67)	1.21 (0.83-1.71)	0.58 (0.35-0.9)	0.5 (0.28-0.81)	0.43 (0.29-0.6)	0.38 (0.26-0.54)
2010	1.66 (0.72-3.27)	0.43 (0.05-1.55)	58.5 (53.65-63.67)	12.33 (10.1-14.9)	138.44 (132.39-144.69)	25.37 (22.72-28.26)	111.39 (102.07-121.33)	21.32 (17.05-26.32)	15.65 (14.23-17.17)	3.44 (2.77-4.23)	1.55 (1.12-2.11)	1.35 (0.94-1.88)	0.71 (0.45-1.06)	0.88 (0.58-1.27)	0.3 (0.19-0.46)	0.41 (0.28-0.57)
2011	2.43 (1.25-4.24)	1.06 (0.34-2.47)	65.69 (60.54-71.16)	13.05 (10.77-15.68)	143.37 (137.17-149.78)	27.32 (24.55-30.31)	109.91 (100.58-119.86)	22.19 (17.85-27.28)	20.3 (18.68-22.03)	4.87 (4.06-5.79)	2 (1.49-2.62)	1.74 (1.26-2.33)	0.91 (0.61-1.31)	1.05 (0.73-1.48)	0.38 (0.26-0.56)	0.51 (0.36-0.69)
2012	1.22 (0.45-2.65)	0.43 (0.05-1.55)	67.38 (62.25-72.82)	13.63 (11.33-16.27)	151 (144.66-157.55)	31.45 (28.5-34.63)	116.96 (107.39-127.15)	21.9 (17.65-26.85)	25.4 (23.59-27.3)	6.09 (5.19-7.09)	2.68 (2.08-3.39)	1.57 (1.12-2.14)	1.89 (1.44-2.43)	1.3 (0.93-1.77)	0.44 (0.3-0.62)	0.52 (0.38-0.7)
2013	1.87 (0.86-3.55)	0 (0-0.81)	68.36 (63.08-73.96)	13.91 (11.54-16.64)	155.66 (149-162.53)	31.69 (28.64-34.98)	125.52 (115.37-136.32)	27.58 (22.67-33.25)	29.28 (27.28-31.39)	7.52 (6.49-8.67)	3.66 (2.94-4.51)	2.01 (1.49-2.67)	2.25 (1.74-2.86)	1.51 (1.1-2.03)	0.56 (0.4-0.77)	0.55 (0.4-0.74)
2014	0.91 (0.25-2.33)	0.48 (0.06-1.73)	72.92 (67.26-78.94)	18.81 (15.93-22.06)	168.06 (160.81-175.54)	34.6 (31.27-38.18)	137.43 (126.36-149.21)	32.11 (26.6-38.43)	34.52 (32.24-36.92)	9.13 (7.95-10.45)	4.8 (3.93-5.82)	2.36 (1.76-3.1)	3.37 (2.71-4.16)	2.05 (1.53-2.68)	0.47 (0.32-0.67)	0.61 (0.45-0.82)
2015	2.77 (1.33-5.09)	0.29 (0.01-1.63)	92.89 (85.88-100.33)	20.7 (17.38-24.47)	187.59 (179.1-196.37)	41.35 (37.34-45.67)	154.49 (141.45-168.39)	37.61 (31.05-45.14)	41.17 (38.42-44.07)	11 (9.56-12.59)	6.54 (5.41-7.83)	3.09 (2.33-4.02)	4.06 (3.24-5.03)	2.29 (1.68-3.05)	0.65 (0.45-0.9)	0.63 (0.44-0.86)
2016	2.64 (1.14-5.2)	0.35 (0.01-1.95)	104.34 (96.43-112.72)	23.41 (19.66-27.68)	198.38 (189.07-208.03)	46.03 (41.52-50.9)	168.79 (154.12-184.49)	38.59 (31.53-46.76)	44.11 (41.05-47.33)	13.93 (12.19-15.85)	7.32 (6.05-8.77)	3.73 (2.84-4.81)	4.21 (3.31-5.29)	3.05 (2.29-3.99)	0.71 (0.49-0.99)	0.71 (0.49-0.98)
2017	1.2 (0.25-3.5)	0.42 (0.01-2.32)	112.13 (103.19-121.64)	21.76 (17.84-26.3)	212.05 (201.65-222.84)	46.58 (41.68-51.89)	158.82 (143.29-175.58)	35.49 (28.19-44.12)	48.51 (44.99-52.22)	13.24 (11.37-15.33)	10.22 (8.6-12.07)	4.72 (3.62-6.03)	4.92 (3.85-6.2)	3.68 (2.76-4.81)	1.19 (0.88-1.58)	0.8 (0.56-1.12)
2018	0.48 (0.01-2.65)	1 (0.12-3.63)	106.2 (96.94-116.09)	28.98 (24.14-34.51)	236.85 (225.4-248.73)	51.75 (46.35-57.6)	160.46 (143.86-178.46)	40.78 (32.53-50.48)	56.25 (52.21-60.53)	16.01 (13.78-18.5)	11.83 (9.95-13.96)	5.94 (4.62-7.52)	6.26 (4.96-7.79)	4.03 (3-5.3)	1.45 (1.09-1.9)	0.99 (0.71-1.35)
