# Supplementary material for: Attention-deficit hyperactivity disorder diagnoses and prescriptions in UK primary care, 2000–2018: population-based cohort study
Source: BJPsych Open. 2023 Jul 17;9(4):e121. doi: 10.1192/bjo.2023.512 (PMC10375867; doi:10.1192/bjo.2023.512)
Supplement: Supplementary file 1 [file bjosup.zip › S2056472423005124sup003.docx]

**Supplementary table 1: List of Read codes and definitions used to determine ADHD diagnoses**

| Read code | Definition |
| --- | --- |
| 9Ngp.00 | On drug ther ADHD (attention deficit hyperactivity disorder) |
| 6A61.00 | Attention deficit hyperactivity disorder annual review |
| 8BPT.00 | Drug therapy ADHD (attention deficit hyperactivity disorder) |
| E2E0z00 | Child attention deficit disorder NOS |
| Eu9y700 | [X]Attention deficit disorder |
| 9Ngp000 | On stim drug ther ADHD (attention def hyperactivity disordr) |
| E2E1.00 | Hyperkinesis with developmental delay |
| ZS93.11 | DAMP - Deficits in attention motor control and perception |
| Eu90111 | [X]Hyperkinetic disorder associated with conduct disorder |
| Eu90z11 | [X]Hyperkinetic reaction of childhood or adolescence NOS |
| ZS94.00 | Minimal brain dysfunction |
| Eu90z00 | [X]Hyperkinetic disorder, unspecified |
| E2E0.00 | Child attention deficit disorder |
| Eu90011 | [X]Attention deficit hyperactivity disorder |
| E2E..11 | Overactive child syndrome |
| ZS91.12 | [X]Attention deficit disorder |
| E2E2.00 | Hyperkinetic conduct disorder |
| ZS91.00 | Attention deficit disorder |
| Eu90200 | [X]Deficits in attention, motor control and perception |
| Eu90100 | [X]Hyperkinetic conduct disorder |
| Eu90.00 | [X]Hyperkinetic disorders |
| ZS91.11 | ADD - Attention deficit disorder |
| Eu90000 | [X]Disturbance of activity and attention |
| E2Ez.00 | Hyperkinetic syndrome NOS |
| Eu90z12 | [X]Hyperkinetic syndrome NOS |
| Eu90y00 | [X]Other hyperkinetic disorders |
| ZS94.11 | MBD - Minimal brain dysfunction |
| ZS93.00 | Deficits in attention motor control and perception |
| E2E0100 | Attention deficit with hyperactivity |
| E2E0000 | Attention deficit without hyperactivity |
| ZS9..00 | Disorders of attention and motor control |
| E2Ey.00 | Other hyperkinetic manifestation |
| E2E..00 | Childhood hyperkinetic syndrome |
